# Mapping Hematopoietic Fate after Transplantation

**DOI:** 10.1007/s12015-025-10946-0

**Published:** 2025-08-20

**Authors:** Stephanie N. Hurwitz

**Affiliations:** 1 Department of Pathology and Laboratory Medicine, Indiana University School of Medicine, Indianapolis, IN USA; 2 Melvin and Bren Simon Comprehensive Cancer Center, Indianapolis, IN USA

**Keywords:** Hematopoietic Stem Cells, Barcoding, Single-cell, Transplantation, Hematopoiesis, Clonal Mapping

## Abstract

**Abstract:**

Hematopoietic stem and progenitor cells (HSPCs) form the foundation of lifelong blood cell production and immune function. Understanding their fate, including how they differentiate, self-renew, and respond to environmental cues has long been a cornerstone of stem cell biology and regenerative medicine. This knowledge is especially vital in the context of therapeutic hematopoietic stem and progenitor cell transplantation, where the diverse behavior of transplanted HSPCs directly impacts patient outcomes. Advances in single-cell omics, lineage barcoding, and in situ tracking now allow us to directly trace the developmental trajectories and clonal contributions of individual HSPCs. These tools are reshaping our understanding of hematopoiesis not as a rigid hierarchy but as a dynamic and adaptive system. This review highlights key technologies that enable fate mapping of HSPCs, integrates insights into clonal behavior during both transplantation and native hematopoiesis, and discusses how these findings are likely to inform future diagnostic and therapeutic strategies.

**Clinical Trial Number:**

Not applicable

**Graphical Abstract:**

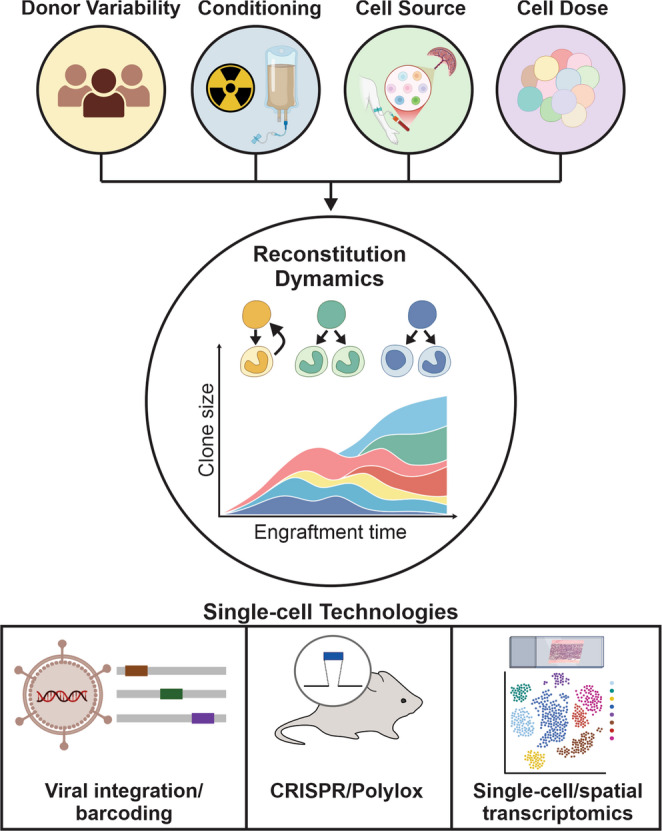

## Introduction

Hematopoietic stem and progenitor cells (HSPCs) sustain lifelong blood and immune system functions, and represent a critical therapeutic tool in the treatment of hematologic malignancies and bone marrow failure syndromes. Allogeneic and autologous HSPC transplantations are potentially curative for a range of disorders, yet clinical outcomes vary widely, with long-term success shaped by the fate of transplanted HSPCs.

Recent advances in lineage tracing, single-cell multi-omics, and spatial technologies have revolutionized our understanding of HSPC biology. In vitro, in vivo, and in silico approaches have revealed extensive behavioral heterogeneity of HSPCs that are marked by variability in self-renewal capacity, clone size, lineage biases, migration patterns, and repopulation kinetics [1–12]. These tools have also revealed that HSPC fate is not solely deterministic. Rather, cells are dynamically regulated by intrinsic transcriptional and epigenetic programs in addition to extrinsic cues from the bone marrow microenvironment. Following transplantation, HSPCs face unique selective pressures, including niche remodeling, immune reconstitution, and therapy-induced stress, that shape their engraftment, clonal expansion, and lineage output. A key insight from work over the past three decades is that hematopoiesis is surprisingly temporally oligoclonal, with evidence of lineage bias, clonal drift, and selective dominance emerging during both normal reconstitution and malignant evolution.

Adding complexity to our understanding that a limited number of dominant clones persist after transplantation is marked donor heterogeneity. Functional differences in donor stem cell output have been observed even among healthy individuals, with barcoding studies in nonhuman primates and humans demonstrating that some donors inherently contribute more balanced, polyclonal, and multilineage reconstitution than others [13–15]. However, few studies have detailed in depth the impact of donor source and mobilization regimens on HSPC pool composition, clonal skewing, and transcriptional heterogeneity [16–18].

Overall, a better understanding of the dynamics of HSPC marrow egress and homing, engraftment, and durable hematopoietic reconstitution will pave the way for optimized transplantation strategies. Knowledge gained from studies into HSPC fate after transplantation may also contribute to improved protocols for ex vivo expansion and gene therapy platforms. Finally, as we move toward clinical translation, how can we harness more sophisticated understanding of repopulation kinetics to predict and prevent poor graft function?

This review will summarize current techniques for mapping HSPC fate after transplantation (Table 1) and synthesize recent insights into how these tools have reshaped our understanding of transplantation biology. By integrating data from experimental models and human studies, we explore how fate mapping is poised to inform clinical decision-making, identify high-risk clones, and ultimately guide therapeutic strategies in hematologic disease.

**Table 1:** Comparison of clonal tracing methods

Feature	Limited Dilution Transplantation	Retroviral Integration Site Analysis	Viral Barcoding	Transposon Tagging	CRISPR/Polylox-based Lineage Tracing
**Core Principle**	Transplanting very few (or single) HSPCs to infer clonal output	Tracking unique retroviral integration sites as clonal marks	Introducing DNA barcodes via viral vectors	Introduction of transposon insertion sites via transposase	CRISPR or Cre-induced mutations or recombinations
**Type of Marking**	Functional (inferred from outcomes)	Genomic (site-specific integration)	Molecular barcode	Genomic (transposon insertion tag)	Regionally accumulated genetic scars
**Bias**	Selection bias for transplantable, active HSCs	Preference for actively cycling cells and transcriptionally active regions	Preference for actively cycling cells and transcriptionally active regions	Semi-random genomic insertion	Some biases in scar generation depending on Cas9 activity
**Scalability**	Low (single/few clones per animal)	Moderate (hundreds of clones)	High (thousands of clones)	High (thousands of clones)	Very high (potentially millions of clones)
**Resolution**	Low	Moderate	High	High (thousands of clones)	Very high
**In Vivo Feasibility**	Requires transplantation	Requires transplantation	Requires transplantation	Native labeling in situ	Native labeling in situ
**Perturbation of Native State**	High (transplantation stress)	High (transduction + transplantation)	High (transduction + transplantation)	Low	Low
**Insertional Mutagenesis Risk**	None	High	Moderate to High	Moderate	Minimal
**Lineage Information**	Yes	Yes	Yes	Yes	Yes
**Single-Cell Compatibility**	No	No	Yes (scRNA + barcode)	Yes (scRNA + tag)	Yes (scRNA + scar analysis)
**Examples of Use**	[9, 19, 20]	[21–31]	[32–34]	[35, 36]	[37–39]

## Techniques for HSPC Fate Mapping

### Single Cell Transplantation

Pioneered in the late 1900s [40], limiting dilution transplantation remains one of the most robust and functionally direct methods for assessing the fate potential of HSPCs [40–42]. This technique involves the transplantation of progressively smaller numbers of phenotypically-defined donor cells, down to a single cell, into lethally irradiated or immunodeficient recipient animals. When successful, single cell transplants can unambiguously link a single HSC to its downstream progeny. These studies have provided crucial insights into clonal heterogeneity, demonstrating that a single HSC can reconstitute multilineage hematopoiesis in mice, and suggesting that mono- or oligoclonality may be a hallmark of long-term reconstitution [1, 7, 9].

Limiting dilution analysis also enables the calculation of stem cell frequency using Poisson statistical models, offering a quantitative estimate of the functional HSC content within a population. Despite the power of this technique, it remains low-throughput and labor intensive. Nonetheless, single-cell and limiting dilution transplantation remain gold-standard methods for validating the developmental potential of HSCs and anchoring newer molecular and in situ fate mapping techniques to a functional framework.

### Viral Integration Site Analysis 

Early studies demonstrating that retroviral vectors could introduce new genetic material into HSPCs paved the way for clonal tracking in hematopoiesis [43–45]. Emerging in part as a means of assessing the safety of viral vectors in gene therapy trials [46–48], integration site analysis has been used by multiple groups to study hematopoietic reconstitution dynamics after transplantation [21–26, 49–51].

In this approach, HSPCs are transduced ex vivo with a replication-incompetent retroviral vector that integrates semi-randomly into the genome of dividing cells. Each integration site acts as a unique and heritable clonal marker, allowing researchers to trace the progeny of a given HSPC after transplantation by identifying the same integration in blood or marrow-derived cells over time.

Integration site retrieval is possible by restriction enzyme digest of genomic DNA that result in fragments containing part of the vector and adjacent genomic DNA. Different integration sites can be detected by Southern blotting [27–29]. More recent approaches amplify fragments by polymerase chain reaction (PCR) followed by sequencing. PCR-based approaches include inverse PCR, where linear fragment ends are self-ligated [53], or ligation-mediated PCR and linear amplification–mediated PCR, where primer tags are attached to fragment ends [52, 54–56]. These strategies provide both qualitative and quantitative readouts of clonal contributions. Fractionation of mature blood cells before digestion also informs lineage bias and relative differentiation capacity of individual HSPC clones [24].

Despite its value, retroviral integration site analysis has limitations. Integration occurs predominantly in dividing cells, possibly excluding the most quiescent long-term HSCs from being labeled. As such, the method may underrepresent the contribution of deeply dormant stem cells. Non-random insertions also pose minimal risk of impacting native HSPC behavior [12, 30, 32, 55, 57–59]. Finally, while PCR-based approaches have increased sensitivity, reliance on restriction enzyme digestions can exclude insertions without appropriately positioned sites, and relative differences in fragment lengths may result in skewed PCR amplification [33]. Nevertheless, retroviral marking remains a foundational tool in HSC biology, particularly in translational contexts where it enables regulatory-grade clonal tracking in gene-modified stem cell products.

### Genetic Barcoding 

In efforts to overcome the limitations of integration site analysis, several groups have utilized a cellular barcoding approach to label clones with a random sequence tag [32–34, 60, 61]. Combined with high-throughput sequencing, DNA barcoding offers a sensitive and powerful tool for tracking individual HSPC clones over time. Initial studies utilized lentiviral delivery of barcode libraries, in which short 20–30 nucleotide sequences are inserted into vector backbones before transduction [32–34, 60, 61]. Libraries are designed to have far more unique sequences than the number of cells to be labeled, ensuring a low probability of multiple cells acquiring the same barcode. To enhance recovery, barcodes are often flanked by common PCR priming sites for later amplification, bypassing the need for restriction digest. Transduction is usually performed ex vivo at a low multiplicity of infection to maintain clonal specificity prior to transplantation.

More recent strategies facilitate endogenous barcoding in vivo. The Polylox murine model, developed by the Rodewald lab, represents a major innovation in fate mapping by allowing in situ generation of high-diversity clonal barcodes without relying on viral transduction or transplantation [37, 38]. At its core, the Polylox system utilizes Cre-mediated recombination between multiple strategically placed loxP sites to generate a large variety of unique genomic rearrangements that can be decoded by sequencing.

In a separate in situ approach, work from the Camargo lab demonstrates the utility of transposon tagging to mark native HSPC clones [35, 36]. This model is based on the premise that temporary induction of a hyperactive Sleeping Beauty transposase mediates quasi-random integration of a cognate DNA transposon into cells, which can serve as a stable genetic tag for a cell and its progeny. An improved method for molecular identification of transposon integration sites using T7-amplification mediated recovery of integration sites (TARIS) has been shown to reduce tag amplification bias [36]. Later, the Camargo lab developed an inducible CRISPR-activated repair lineage tracing (CARLIN) model [39, 62]. CARLIN mice contain Cas9, guide RNAs, and a barcode that comprises ten different guide RNA sites. Following Cas9-mediated editing in the barcode region, semi-random insertions and deletions are created by native DNA repair mechanisms, acting as a unique and heritable cellular tracer.

One of the major advantages of genetic barcoding over earlier methods like retroviral integration site analysis is the sheer number of clones that can be tracked simultaneously, ranging from hundreds to thousands per experiment. This has enabled a detailed view of the heterogeneity and temporal reconstitution of HSPCs, with recent innovations in in situ labeling highlighting crucial insights into native versus regenerative dynamics (Table 1).

### Multi-Omic Technologies

Over the past decade, single-cell RNA sequencing (scRNA-seq) and related multi-omic approaches such as single-cell Assay for Transposase-Accessible Chromatin with sequencing (ATAC-Seq) and Cellular Indexing of Transcriptomes and Epitopes by sequencing (CITE-seq) have uncovered striking transcriptional heterogeneity within HSPC compartments [63–74]. Downstream computational approaches, including pseudotime analysis and trajectory inference now allow in silico reconstruction of lineage decisions that have highlighted lineage commitment along a continuum [64, 68, 69, 75–78]. When paired with clonal markers or lineage barcodes, these data can provide multi-faceted insights into the molecular programs guiding HSPC fate [36, 78].

### Spatial Mapping

Intravital microscopy has enabled real-time visualization of HSPC behavior in bone marrow niches, providing insights into stem cell localization, motility, and division patterns [79, 80]. Demonstrating the utility of integrating spatial data and scRNA-seq, more recent studies have adapted these technologies to detail cellular interactions and signaling gradients in the bone marrow. In situ localization of bone marrow cells has been captured through laser-capture microdissection coupled with sequencing [81] or co-detection by indexing (CODEX) [82]. Emerging technical and analytical innovations have further addressed historic limitations of processing mineralized bone marrow tissue for spatial transcriptomics, and detailed hematopoietic and non-hematopoietic cellular interactions in the native murine and human bone marrow microenvironment [83–85]. In the future, combined single-cell and spatial transcriptomics are poised to address key questions surrounding the microenvironmental regulation of transplanted HSPCs, including deconvoluting clonal behavior and niche localization.

## Key Insights into Hematopoiesis from Tracking HSPC Fates

### A Small Fraction of the HSPC Pool is Likely Mobilized from Donors

Several techniques, including limiting-dilution transplantation assays [86], somatic mutation-based phylogenetic reconstruction [87, 88], and fluorescence-based binomial distribution modeling [89, 90] have been used to quantitate the pool of native, active HSPCs in mice and humans with most estimates ranging from 10,000 to 200,000 in healthy individuals [87–89]. In one study by Mitchell et al., aged adults (75 years and older) showed profoundly decreased clonal diversity, with an estimated 12–18 independent clones actively contributing to the majority of hematopoietic output [88].

Mobilization with granulocyte colony-stimulating factor (G-CSF), often in combination with CXCR4 antagonists like plerixafor, releases a heterogeneous population of hematopoietic progenitors, of which true long-term HSCs (LT-HSCs) represent a minor component [18]. Mobilization tends to favor certain subsets of progenitors; for example, myeloid-biased HSPCs may preferentially egress, while more quiescent, LT-HSCs may be underrepresented. Unpublished data from our lab has suggested that immunophenotypically defined LT-HSCs (Lin- CD19- CD34 + CD38- CD45RA- CD90 + CD49f+) make up between 0.003 and 0.02% of mobilized CD34 + cells. Combined with prior functional studies suggesting approximately 3 of 10 CD49f + HSCs engraft from cord blood [91], and knowing that most patients undergoing transplantation receive 2.5–10 × 10^6^ CD34 + cells/kg, these findings suggest that between roughly 1,500 − 45,000 functional LT-HSCs may be routinely engrafted in patients.

The mechanisms underlying selective mobilization remain incompletely understood but may involve differential niche anchorage, expression of adhesion molecules (e.g., VLA-4, CXCR4), and intrinsic differences in cycling or quiescence status [92, 93]. Studies in mice and humans have suggested some degree of stochasticity in mobilization between clones, implying that altogether HSPC migration may be probabilistic [16, 94]. Further investigation is needed to assess putative intrinsic differences in stem cell pools collected by various mobilization strategies, or within cord blood units. These findings will likely have direct implications for transplant outcomes, and underscore the importance of understanding and optimizing mobilization protocols to sustain long-term hematopoiesis.

### Homing to the Bone Marrow is Relatively Inefficient

After intravenous infusion, HSPCs must home efficiently to specialized niches within the bone marrow to successfully engraft and reconstitute hematopoiesis. This rapid multistep process takes place within minutes to hours and begins with HSPCs trafficking through the circulation and tethering to the bone marrow vasculature, primarily the sinusoidal endothelium [95]. Selectins mediate the initial rolling interactions: P-selectin and E-selectin, expressed by bone marrow endothelial cells, interact with ligands on HSPCs such as P-selectin glycoprotein ligand-1 (PSGL-1) and CD34 [96, 97]. Firm adhesion is then mediated by integrins, especially VLA-4 (α4β1 integrin) binding to VCAM-1 (vascular cell adhesion molecule-1) on endothelial cells [96, 98–100]. Following adhesion, HSPCs undergo transendothelial migration into the marrow parenchyma, a process facilitated by chemokine signaling, particularly the CXCL12/CXCR4 axis [99–101]. Additional molecules, including sphingosine-1-phosphate [102], extracellular nucleotides [103], and CCR2 ligands [104] also modulate homing efficiency. In the fetal liver, HSPC colonization triggers physical adaptation of ECs to sustain HSPC occupancy in a term called “cuddling” [80]; however, these interactions in the adult bone marrow have not been detailed. Within the marrow, HSPCs interact with a variety of niche cell types, including mesenchymal stromal cells, endothelial cells, osteoblasts, and CXCL12-abundant reticular cells which further regulate their lodgment, survival, and initiation of hematopoiesis [105].

Homing efficiency is a critical determinant of transplant success and may be influenced by factors such as inflammation, conditioning regimens, and donor cell manipulation. Best estimates from single-cell transplantation experiments across several labs suggest engraftment rates into irradiated mice to be 10–35% [106–111], with a single study demonstrating substantially higher engraftment [112]. In vivo bioluminescence imaging of transplanted HSPCs corroborated these initial reports, demonstrating that the number of donor cell foci in recipients was substantially smaller than the number of transplanted HSPCs [113]. These findings suggest that a limited number of niches support HSPC engraftment and expansion, or that seeding of HSPCs into competent microenvironments is limited by intrinsic cell properties.

Despite these predictions, precise measurement of initial bona fide marrow homing has been challenging to disentangle from short and long-term engraftment. Reliance on long-term functional reconstitution studies from single cell transplantation may underestimate numbers of homed, short-lived HSPCs. In contrast, numerical assessments of marrow HSPCs at early timepoints after transplantation may be overestimated, as some studies have shown rapid HSPC division as early as the first day after transplantation [114].

Adding complexity are described impacts of donor cell source on homing efficiency. Mobilized peripheral blood CD34 + cells harbor increased selectin-mediated rolling, adhesion, and transmigration compared to umbilical cord blood cells [115], which may contribute to faster early engraftment. Use of G-CSF in combination with plerixafor, a CXCR4 antagonist, in mobilization regimens leads to earlier neutrophil and platelet engraftment compared to G-CSF alone, independent of cell dose [116, 117]. Whether this observation is due to mobilization of select precursors harboring distinct engraftment properties, or reprogramming of an analogous HSPC pool is uncertain.

### Reconstitution Dynamics are Tightly Linked to Transplantation Cell Dose 

Intuitively evident from murine models and clinical studies is that transplanted cell dose is a critical determinant of engraftment success, hematopoietic recovery kinetics, and long-term clonal architecture. In murine models, limiting dilution transplantation studies have confirmed that reconstitution capacity scales proportionally with the number of functional HSCs transplanted [34, 40, 42]. In patients, higher doses of CD34⁺ cells (above 5 × 10⁶ CD34⁺/kg) are consistently associated with more rapid neutrophil and platelet recovery, lower rates of graft failure, and improved survival outcomes [118–120]. In work by Six et al., a patient engrafted with a very high dose (13.6 × 10⁶ CD34⁺/kg) was estimated to harbor 50,000 clones contributing to hematopoiesis [24]. Taken together with recent estimates of the number of active HSPCs in healthy individuals (10,000-200,000) [87–89], these data suggest likely clonal bottlenecks in lower doses.

At very low doses, hematopoiesis becomes oligoclonal or monoclonal, with a few engrafting clones dominating. Given the heterogeneity in contribution of individual HSC clones to blood cell supply [19, 32, 58, 121, 122], these clonal restrictions introduce risks of lineage skewing, stochastic graft failure, or insufficient long-term repopulation. High-dose transplants, by contrast, not only promote rapid hematopoietic recovery but also preserve clonal diversity [23, 24]. Dose-dependent changes in HSC differentiation programs have also been observed [34]. At high transplantation doses, donor clones are more likely to differentiate early and produce all cell types later, while low doses lead to larger proportions of clones appearing later and producing more specialized cell types.

### Early Reconstitution After Conditioning is Marked By Clonal Fluctuation

**Fig. 1:**
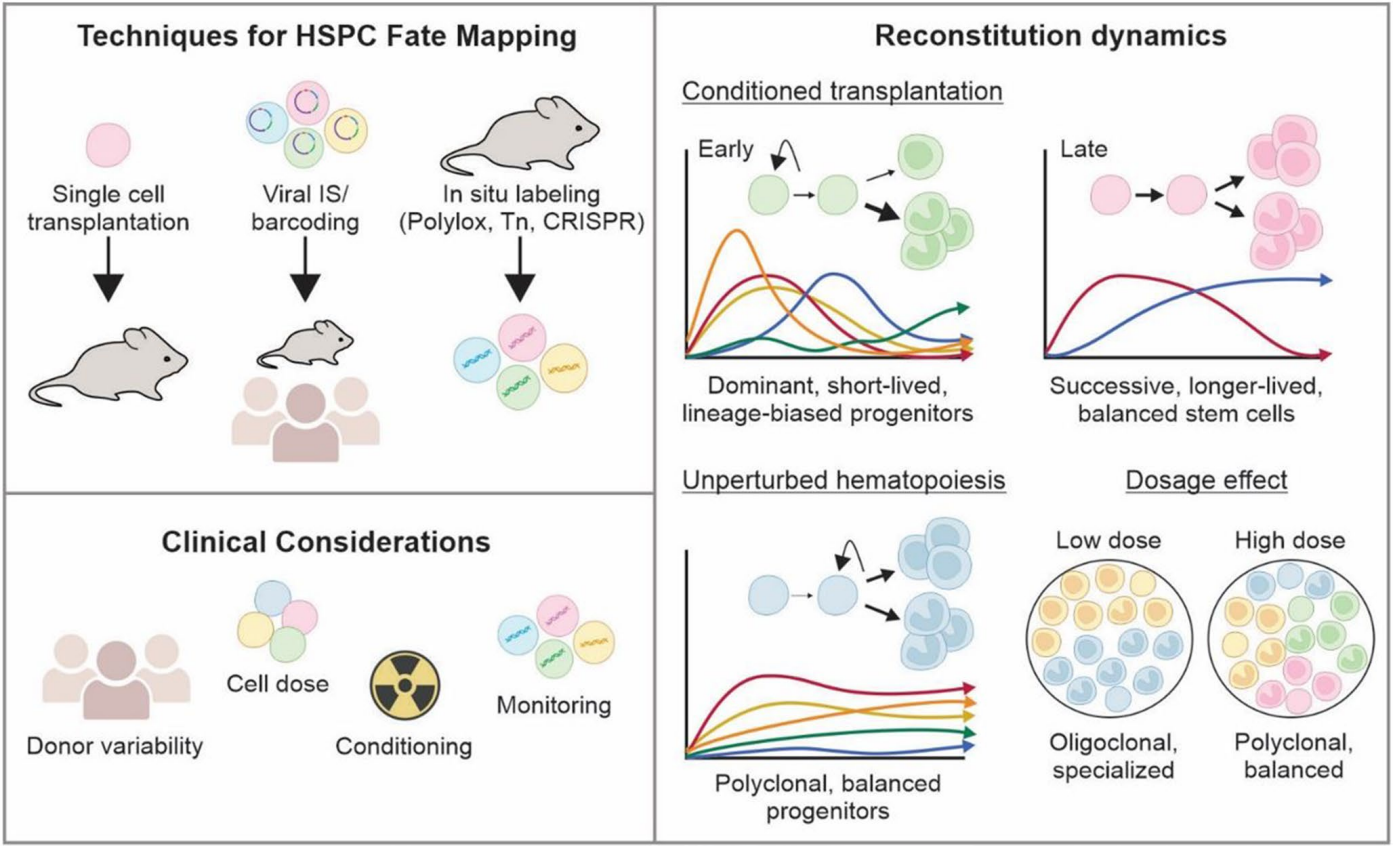
Summary of HSPC clonal tracking techniques, key insights, and clinical considerations

Through integration site and barcoding experiments, key insights into the early kinetics of HSPC engraftment following transplantation have emerged. These studies have revealed striking patterns of temporal clonal expansion and restriction, with the following common themes (Fig. 1):

*HSC self-renewal occurs early after conditioned transplantation.* Consistent with the expectation that donor HSCs must expand via self-renewal to reconstitute the entire HSC pool, Lu et al. demonstrated that by week 22 after conditioned transplantation, HSCs had undergone at least three cycles of self-renewal [61].

*Early HSPC clones show heterogeneous lineage bias.* Early post-transplant reconstitution is predominantly driven by a broad pool of progenitor-like clones with lineage-restricted output. Confirmation by several groups has revealed myeloid-biased (My-Bi), balanced (Ba) and lymphoid-biased (Ly-Bi) HSCs that arise from dominant differentiation at distinct lineage commitment steps [5, 20, 24, 26, 32, 34, 61, 121–123]. Growing evidence also supports the identity of a platelet-biased HSC that may contribute to rapid platelet production during emergency hematopoiesis and inflammation [36, 124, 125]. Lineage biases of donor-derived clones may be in part dependent, or overcome by, transplantation cell dosage [32, 34, 61]. In clinical contexts, lineage skewing can have significant implications for infection risk, graft-versus-host disease (GVHD), and relapse.

*Initial reconstitution is marked by high numbers of short-lived clones.* Longitudinal functional and clonal tracking studies have demonstrated that early reconstitution is dominated by a large number of short-lived progenitor-dominated clones that fluctuate over time, with limited transplantation capacity [1, 4, 21, 25, 26, 29, 31, 32, 35, 61, 126, 127]. These early clones may persist or be replaced by more stable long-term clones. Altogether, a common theme observed is a reduction or extinction of some clones, accompanied by an increase in other clones; this has been termed clonal equilibration [1]. Notably, the fraction of donor-derived clones giving rise to each cell type is inversely correlated with the transplanted cell dose, supporting a limit to engraftment that may be due in part to competent niche availability and cell-cell competition [34, 128].

### Long-term Hematopoiesis is Stabilized By a Small Number of Balanced, Sequential Clones

As hematopoiesis stabilizes, a smaller subset of clones begins to predominate. Several studies have indicated that long-term repopulating clones arise gradually, with clonal fluctuation stabilizing at about 1 year post-transplant [4, 21, 25, 26]. Long-term maintenance is strikingly oligoclonal, with a small number of clones contributing to the majority of total graft output [1, 4, 32, 126]. While lineage-biased subsets persist at later timepoints after transplantation [25], the greatest proportion of repopulation is ultimately contributed by the balanced type clones [1, 26, 32]. That said, this balanced model may vary across disease types; in particular, primary immunodeficiency patients treated with gene therapy have been observed to harbor long-lived lymphoid-skewed clones [25]. Finally, even the most stable clones appear to have a finite lifespan. In a phenomenon termed “clonal succession,” sequential declines in clones are compensated by expansions of others, together maintaining a stable hematopoietic output for years post-transplantation [4, 26, 29, 31].

### Repopulation dynamics are distinct in an unperturbed niche

Studies tracking HSPC behavior in unconditioned transplantation or in native bone marrows have revealed dramatically different dynamics. While absolute numbers of engrafted cells are higher in the presence of conditioning strategies such as total body irradiation (TBI), niche perturbations create a pro-inflammatory milieu that drives transplanted HSPCs into early proliferation and hematopoietic contribution. In contrast to the lineage-biased, short-lived oligoclonal populations seen during reconstitution in mice after TBI, donor-derived clones after unconditioned transplants uniformly differentiate and self-renew, contributing homogeneously to granulocytes and B-cells over time [65]. In sum, clonal dominance is minimal, and reconstitution remains polyclonal. In addition, HSC-harbored barcodes are not significantly amplified in mice after unconditioned transplantation, indicating an absence of clonal stem cell expansion. Despite otherwise uniform lineage commitment, it has been noted that equal contribution to T-cells is not observed; this finding may in part be explained by thymic maturation and restriction [129].

In situ clonal tracking, including through Polylox, CRISPR, and transposon tagging have additionally shed light on physiologic hematopoiesis, allowing lineage tracing within the native bone marrow in the absence of niche disruption and transplantation stress. In contrast to the post-transplantation setting where later stable, dominant peripheral blood clones originate from LT-HSCs, progenitors play a central role in unperturbed hematopoiesis with minimal and gradual contribution from LT-HSCs [35, 39, 130]. Interestingly, a large majority of LT-HSC clones may contribute exclusively to direct megakaryopoiesis [36]. In mice, low flux from LT-HSCs in situ may be explained in part by large estimated reservoirs of ST-HSCs that are nearly sufficient to compensate for steady-state cell loss [35, 130]. As such, long-term steady-state granulopoiesis is greatly polyclonal, and may be driven by both simultaneous and successive recruitment of non-overlapping clones.

While early in development unilineage progenitors dominate, many later subsets of HSCs (including embryonically-marked precursors) contribute consistently to long-term, multilineage hematopoiesis [36, 37]. Myelo-erythroid biased or restricted HSCs, or those that lack lineage output altogether (“differentiation-inactive”) have been described [38]. However, clonal origins of granulocytes and monocytes are tightly linked under both steady state and transplantation conditions [35, 127], and in post-developmental stages, lymphoid biased or restricted clones are rare [35]. Finally, combined with single-cell gene expression profiling, these studies show that coherent fates of HSCs in a clone are rooted in similar transcriptomes [38].

Together, these biologic tools offer approaches that can begin to tackle open questions, including to what extent the dramatic alterations in post-transplant clonal kinetics are due to regenerative demand versus damaged niche remodeling. One piece of evidence arguing a significant local microenvironmental effect is the observation that fetal liver-derived HSCs display heterogeneous expansion potentials conferred by the bone site into which they home [39]. These findings argue for additional consideration in optimizing conditioning strategies for transplant patients.

## Clinical Considerations

While clinical practice has long recognized the importance of factors such as donor matching, cell dose, and conditioning intensity, clonal tracking studies now provide mechanistic context for how these parameters affect long-term engraftment, lineage output, and overall patient outcomes.

*Cell dosing*. One key consideration is HSPC dose, which directly influences not only the kinetics of repopulation, but also clonal diversity and multilineage output. As advances in mobilization strategies and ex vivo expansion take shape and allow for greater numbers of transplantable cells, these considerations may raise the acceptable minimal or standard dosing in high-risk patients.

*Donor selection*. Donor variability has also emerged as a crucial determinant of outcome. Even among HLA-matched donors, intrinsic differences in HSPC composition, cycling status, and lineage bias can affect both the pace and quality of engraftment. As we continue to understand epigenetic regulation of HSPCs, particularly inflammatory memory, infection or vaccination history may serve a role in donor selection. Further phenotypic and functional characterization of differentially mobilized donor cells may also pave the way for optimized collection strategies. The sum of donor variability further underscores the potential value of functionally characterizing grafts prior to infusion.

*Conditioning*. The conditioning regimen is another key modulator of HSPC behavior. While myeloablative therapies maximize niche availability and facilitate rapid engraftment, they may also select for progenitor-dominant clones with limited durability. Reduced-intensity or unconditioned approaches preserve host niche architecture but require greater competitive fitness from donor HSPCs and may lead to chimerism or mixed lineage output. Understanding how different conditioning environments shape HSPC clonal dynamics is essential for tailoring transplant strategies to specific disease contexts. This knowledge may also inform more targeted, non-toxic conditioning approaches.

*Clonal tracking.* HSPC fate mapping currently offers a powerful tool for monitoring therapeutic safety and efficacy in the setting of gene therapy. Assessment of clonal diversity holds further promise in all allogeneic or autologous transplants, where novel clonal tracking technologies may support personalized approaches to stem cell therapy and immune reconstitution, including through improving risk stratification, transplant design, and graft monitoring.

*Large data modeling.* Finally, the integration of artificial intelligence tools will likely accelerate clinical translation of growing molecular insights in transplantation medicine. Traditionally, prognostic markers of graft success or GVHD risk have relied on limited parameters such as early post-transplant clinical signs, biopsy findings, chimerism levels, or single biomarkers [131, 132]. As we move forward, computational modeling using growing multi-omics data generated by HSPC mapping studies may uncover latent variables predictive of durable engraftment, lineage bias, or disease relapse. Application of spatial mapping strategies to routine pre- and post-transplantation bone marrow biopsies could usher in a new age of personalized conditioning regimens, donor selection, or post-transplant surveillance strategies.

## Conclusions

These technologies have converged on several key principles. First, native long-term hematopoiesis is maintained by a relatively small pool of balanced clones that arise gradually and persist over time. Second, transplantation itself imposes a distinct physiologic state that biases early hematopoiesis toward transient, progenitor-like clones, contrasting with the quiescent, self-renewing activity of endogenous HSCs. Third, donor variability, cell dose, conditioning regimen, and niche integrity all influence HSPC behavior post-infusion, with implications for transplant success, clonal dominance, and relapse in disease contexts such as leukemia. Emerging evidence supporting epigenetic memory in HSCs raises the possibility of additional unexplored donor cell variability that may contribute to stem cell plasticity or predetermined behavior [5, 133, 134].

Understanding the fate and kinetics of individual HSPCs has direct clinical relevance, not only for optimizing donor cell collection and transplantation protocols, but also for predicting graft durability, interpreting clonal hematopoiesis, improving gene therapy vector safety, and designing next-generation regenerative and immunologic therapies. As lineage tracing tools continue to advance in resolution and scalability, future studies integrating single-cell multi-omics, spatial localization, and long-term clonal history will be essential to fully elucidate the rules governing stem cell fate in health and disease.

## Data Availability

No datasets were generated or analysed during the current study.
